# Structural Equation Modeling for Analyzing Erythrocyte Fatty Acids in Framingham

**DOI:** 10.1155/2014/160520

**Published:** 2014-04-15

**Authors:** James V. Pottala, Gemechis D. Djira, Mark A. Espeland, Jun Ye, Martin G. Larson, William S. Harris

**Affiliations:** ^1^Health Diagnostic Laboratory Inc., Richmond, VA 23219, USA; ^2^Department of Internal Medicine, Sanford School of Medicine, University of South Dakota, Sioux Falls, SD 57105, USA; ^3^Department of Mathematics and Statistics, South Dakota State University, Brookings, SD 57007, USA; ^4^Department of Biostatistical Sciences, Wake Forest School of Medicine, Winston-Salem, NC 27157, USA; ^5^Department of Statistics, University of Akron, Akron, OH 44325, USA; ^6^Department of Biostatistics, Boston University School of Public Health, Boston, MA 02218, USA; ^7^Department of Mathematics and Statistics, Boston University, Boston, MA 02215, USA; ^8^Framingham Heart Study, Framingham, MA 01702, USA; ^9^OmegaQuant Analytics, Sioux Falls, SD 57107, USA

## Abstract

Research has shown that several types of erythrocyte fatty acids (i.e., omega-3, omega-6, and *trans*) are associated with risk for cardiovascular diseases. However, there are complex metabolic and dietary relations among fatty acids, which induce correlations that are typically ignored when using them as risk predictors. A latent variable approach could summarize these complex relations into a few latent variable scores for use in statistical models. Twenty-two red blood cell (RBC) fatty acids were measured in Framingham (*N* = 3196). The correlation matrix of the fatty acids was modeled using structural equation modeling; the model was tested for goodness-of-fit and gender invariance. Thirteen fatty acids were summarized by three latent variables, and gender invariance was rejected so separate models were developed for men and women. A score was developed for the polyunsaturated fatty acid (PUFA) latent variable, which explained about 30% of the variance in the data. The PUFA score included loadings in opposing directions among three omega-3 and three omega-6 fatty acids, and incorporated the biosynthetic and dietary relations among them. Whether the PUFA factor score can improve the performance of risk prediction in cardiovascular diseases remains to be tested.

## 1. Introduction

Higher blood levels of the essential omega-3 polyunsaturated fatty acids (PUFA) are associated with reduced risk for sudden cardiac death [1, 2] and all-cause mortality [2, 3]. There is also evidence that the essential omega-6 PUFA intakes and blood levels are inversely associated with risk for coronary heart disease [4]. Other fatty acids, such as the* trans* fatty acids found in partially hydrogenated vegetable oils, are believed to increase risk for cardiovascular disease [5]. Hence, the study of these fatty acids is of vital importance.

**Figure 1:**
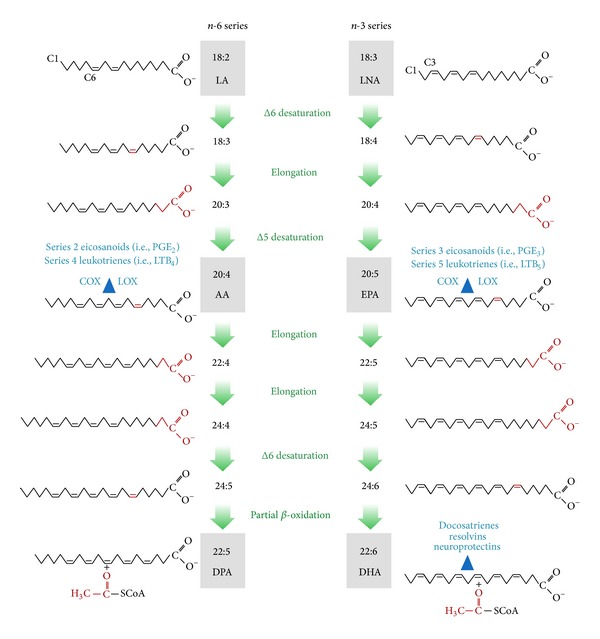
Polyunsaturated fatty acid biosynthesis [7]. Permission to reproduce this figure was granted on December 26, 2013, from the journals' copyright clearance center. Biosynthesis of long-chain* n-3* and* n-6* series polyunsaturated FAs from their 18-carbon precursors. The terminal methyl group is carbon 1 and the* n-3* and* n-6* series of FAs are termed according to the position of the first double bond: after carbon 3 and carbon 6, respectively. Biologically important FAs are highlighted with a gray box. Newly added/removed carbons or double bonds introduced at each step are colored red. Signaling molecules derived from AA, EPA, and DHA are noted in blue. LA, linoleic acid; LNA, linolenic acid; AA, arachidonic acid; EPA, eicosapentanoic acid; DPA, docosapentanoic acid; DHA, docosahexaenoic acid; COX, cyclooxygenase; LOX, lipoxygenase; PG, prostaglandin; and LT, leukotriene.

PUFA are “essential” since they cannot be produced* in vivo *and must be consumed. Foods are composed of multiple fatty acids, and dietary habits manifest themselves as correlated fatty acid levels in the blood. Once consumed, simpler PUFA species can be acted upon by enzymes that convert them into more complex PUFA which have a wide variety of metabolic functions. Desaturase enzymes insert double bonds (points of “desaturation”) into fatty acid molecules, and elongase enzymes are needed to increase the carbon chain length [6]. Importantly, competition among PUFA species exists for these enzymes such that different ratios in the diet can affect overall PUFA patterns (Figure 1) [7]. While elongase enzymes are readily available, the desaturase enzymes are rate limiting, and thus their levels may impact the amount of the 20- and 22-carbon fatty acids present in the system [8]. Additionally, omega-3 fatty acids are the preferential substrates over omega-6 for these desaturase enzymes [6]. These biochemical and dietary relations induce a correlation structure in the blood fatty acids.

Fatty acids have been reported as weight%, mol%, or concentration (by volume or cell count). Since there are no laboratory standards in the USA to uniformly report fatty acid data, these multiple presentations exist. The main debate is relative versus absolute amounts, whose quantities become more divergent as they increase [9]. Chow argues in favor of absolute concentrations, citing the obvious drawback of relative amounts being the imposed linear constraint (i.e, summation to 100%) [10]. However, Crowe prefers relative amounts since absolute concentrations rise and fall with total cholesterol, which is made up of lipoproteins composed of fatty acids [11]. Fatty acid nomenclature is as follows: C#:#n# = the number of carbon (C) atoms in the molecule, the number of double bonds, and the omega family (n), whether 3, 6, 7, or 9. The latter indicates on which carbon the final double bond resides. In Bradbury et al. concentrations and mol% are compared for C14:0 and C18:2n6 in plasma cholesterol ester and phospholipids [12]. The study results show that C18:2n6 is significantly* directly* correlated with total cholesterol when represented as a concentration (*μ*mol/L) but significantly* inversely* related as mol%. The paper lists several references that support the cholesterol lowering effect of C18:2n6 and concludes that the metabolic pathways are influenced by the percentage of total fatty acids and not by concentration. The other advantage of weight% representation is that RBC fatty acids have a strong correlation with myocardium tissue fatty acids (*r* = 0.82) [13] and dietary intake [14]. This preferred technique of using relative weight% of total fatty acids also induces a correlation structure in the data.

Structural equation modeling (SEM) is well suited to incorporate the metabolic, dietary, and measurement correlations observed in fatty acid data. Only in the last decade has SEM been applied to fatty acids [15–18]. SEM allows complex high-dimensional relations to be simplified into a few latent variable scores, which can be evaluated as novel risk markers. Sex-specific risk prediction models have been implemented for coronary heart disease [19] to account for gender differences in the amount of risk attributable to cholesterol and blood pressure levels. Similarly the observed fatty acids may relate to the underlying latent constructs differentially for men and women, and this potential gender invariance needs to be evaluated. A recent taxonomy has been developed to specifically test measurement invariance over multiple groups in SEM by comparing models with different constraints applied to the correlation structure [20, 21].

The objective of the present study was to reduce the dimensionality of the complex fatty acid correlation structure by incorporating dietary intake patterns and biosynthesis processes as constraints in a structural equation model. This technique was applied to the Framingham Offspring/Omni RBC samples, and differences in fatty acid means, loadings, residuals, and latent variable covariance structures between men and women were tested.

## 2. Materials and Methods

### 2.1. Materials

The Framingham Heart Study (FHS) was established in 1948 to research the factors that contribute to cardiovascular disease. Its study design and methods are described at http://www.nhlbi.nih.gov/about/framingham. In 1971, the children (and their spouses) of the original FHS were recruited; they constitute the Framingham Offspring cohort [22]. In 1994, to better reflect the changing demographics of the area, recruitment began for Framingham residents aged 40–74 who described themselves as members of a minority group, that is, Omni cohort [23]. The Offspring and Omni cohorts were scheduled together for comprehensive examinations every 4–8 years. These included anthropometric measurements, biochemical assessment for CVD risk factors, medical history, and physical examination by a study physician. RBC samples taken from Offspring Examination 8 and Omni Exam 3 (2005–2007) were collected and subsequently 22 fatty acids were analyzed using gas chromatography (GC), and their content was expressed as a weight% of total fatty acids [24]. These data are publicly available as part of the National Heart Lung and Blood Institute (NHLBI) SNP Health Association Resource (SHARe) project (release date: March 26, 2013, dataset name: l_rbcfa_2008_m_0420s, dataset accession: pht002568). Written informed consent was provided by all participants, and the Institutional Review Board at the Boston University Medical Center approved the study protocol.

The study participants had a mean age (SD) of 66 (9) years, 55% were female, and 91% were white (see Table 1in Supplementary Material available online at http://dx.doi.org/10.1155/2014/160520). The prevalence of chronic disease was diabetes (14%), heart disease (10%), and congestive heart failure (2%). The participants were taking hypertension medications (49%), lipid pharmacotherapy (43%), aspirin 3+ per week (43%), and fish oil supplements (10%).

### 2.2. Methods

#### 2.2.1. Model Notation

SEM is a two-part modeling process. The first part defines a measurement model, which specifies the relations between the fatty acids and the latent variables, given by **y**_*i*_ = ***ν*** + Λ**η**_*i*_ + **ε**_*i*_ (*i* = 1,…, *N*) [25] where **y**_*i*_ is a *p* × 1 vector of observed fatty acids measured on subject *i*, ***ν***  is a vector of fatty acid means, Λ is a matrix of unknown loading parameters, **η**_*i*_  is a *m* × 1 vector of latent variable scores for subject *i*, and **ε**_*i*_ is vector of normal random errors with covariance matrix Θ that is independent of **η**_*i*_. The second part defines a path model for the latent variables **η**_*i*_, which allows regressing one latent variable **η**_1*i*_ on the set of other latent variables **η**_2*i*_, given by **η**_1*i*_ = **α** + **B** **η**_2*i*_ + **ξ**_*i*_, where **α** is a vector of latent variable means, **B** is a vector of unknown regression parameters, and **ξ**_*i*_ is vector of normal random errors with covariance matrix Ψ.

#### 2.2.2. Comparing SEM to Other Multivariate Techniques

The proportion of variance explained in the *j*th fatty acid by the latent variables is defined as the *j*th communality. The variance in each fatty acid is equal to its communality plus its unique variance; that is, *σ*_*jj*_ = *λ*_*j*1_^2^ + *λ*_*j*2_^2^ + ⋯+*λ*_*jm*_^2^ + *θ*_*j*_. Principal components analysis (PCA) ignores the specific variance (measurement error) and uses the identity matrix for *θ*_*j*_. This results in factoring the* total* variance instead of the* common* variance; the latter is the proportion of variance shared by the fatty acids. If communalities < 1 are used with principal components method of decomposing the observed correlation matrix using eigenvalues and eigenvectors, then the method is principal factoring. This is akin to using the reduced sample correlation matrix where the main diagonals are less than one. Likewise, an exploratory factor analysis imposes no structure and also assumes independence in the residuals matrix, but the common variance is extracted and the factors can be correlated through oblique rotations. Moving to confirmatory factor analysis requires restrictions on the model parameter, which allows testing the model goodness-of-fit. However, only structural equation modeling (SEM) allows regression paths among the observed and unobserved variables, also a sparse correlation matrix for the residuals can be specified. Therefore, SEM allows the most model flexibility for implementing the dietary intake patterns and metabolic processes among the fatty acids.

#### 2.2.3. Data Preparation

SEM requires multivariate normality for maximum likelihood (ML) estimation. We started by assessing univariate normality which is implied by multivariate normality. To examine univariate normality, skewness and kurtosis measures were calculated for each fatty acid. If a fatty acid had an absolute kurtosis >10, considered problematic [26], or skewness >3, then a natural logarithm transformation was employed. Next, the null hypothesis of multivariate normality was tested using the SAS macro %MULTNORM which calculates the squared Mahalanobis distances (*D* ^2^); for large samples, *D* ^2^ is distributed as a *χ*_*p*_^2^ [27]. When multivariate normality is not tenable, robust ML estimation should be implemented [28].

The scales of the RBC fatty acids differed by two orders of magnitude, on average Palmitic acid (C16:0) accounted for 20% of total fatty acid abundance, whereas alpha-linolenic acid (C18:3n3) accounted for only 0.2%. However, there are meaningful fatty acids even at small relative weight%. For example, the mean levels of C20:5n3 and C16:1t are 0.7% and 0.2%, but these are widely studied biomarkers of fish and dairy intake, respectively. Therefore, all fatty acids were standardized in order to have similar effect sizes, which prevented the large abundance fatty acids from dominating the variance extraction. The measure of sampling adequacy developed by Kaiser [29] MSA = Σ(simple  correlations)^2^/[Σ(simple  correlations)^2^ + Σ(partial  correlations)^2^] was used to identify fatty acids that were not sufficiently related to the core latent structure. Individual fatty acids with a MSA <0.60 were considered “unacceptable” [30] and dropped from analysis.

#### 2.2.4. Gender Invariance Testing

The primary motivation of the study was to reduce the dimensions of the fatty acid correlation matrix and to develop latent variable scores for each subject using the regression scoring method [31]. An assumption when using latent variable scores is that the indicators (i.e, fatty acids) have the same relations with the underlying latent variables between groups of interest, in this case gender. To this end, the correlation matrix was partitioned as Σ = ΛΨΛ^**T**^ + Θ, and it along with the mean profile ***ν*** of the observed fatty acids was tested for gender invariance using the likelihood ratio test (LRT) by comparing models with different imposed constraints. When using robust maximum likelihood the LRT has been modified by the deviance scaling [32]. The specific hypotheses are as follows.

1. The fatty acid means are equal between genders, H_1_ : ***ν***_male_ = ***ν***_female_.
2. The loadings matrix is equal between genders, H_2_ : Λ_male_ = Λ_female_.
3. The fatty acid variances are equal between genders, H_3_ : Θ_male_ = Θ_female_.
4. The latent variable covariances are equal between genders, H_4_ : Ψ_male_ = Ψ_female_.
5. The fatty acid covariances are equal between genders, H_5_ : Σ_male_ = Σ_female_.

The number of underlying dimensions was examined using exploratory factory analysis where eigenvalues >1 were retained. Then a SEM was built using the same number of latent variables, and it was used to test if the fatty acid means ***ν*** were equal between men and women (H_1_). This was done by testing the model *X* ^2^ between a model with intercepts, loadings, and unique variances freely estimated for men and women (model M0) versus one with a single set of intercepts imposed for both genders (model M1). To test for equality in the loading matrix Λ between genders (H_2_), a model with equal loading constraints, but freely estimated intercepts and unique variances for each gender (model M2), was compared to model M0. Hypothesis H_3_ was tested for gender differences in fatty acid residual variances Θ by comparing a model with freely estimated intercepts and loadings for each gender, but with constrained variances (Model M3) versus model M0. To test the latent variable covariance structure Ψ, model M2 was used for comparison with additional constraints placed on the six latent covariances to be equal between genders (Model M4). Lastly the fatty acid covariance matrix was tested by constraining loadings, latent covariances, and unique variances to be equal for men and women (Model M5). Each model used the direct Quartimin oblique rotation (available in Mplus and SAS).

#### 2.2.5. Comparing Model Fits

To evaluate the fit of the SEM there are several indices, and the following is the minimal set established by current practice: (1) model chi-square, (2) Steiger-Lind root mean square error of approximation (RMSEA), (3) Bentler comparative fix index (CFI), and (4) standardized root mean square residual (SRMSR) [26]. The RMSEA indicates the discrepancy in model fit per degree of freedom as defined by *ε* = sqrt[(*χ* ^2^ − df)/(df × (N − 1))] [33]. The RMSEA follows a noncentral *X* ^2^ distribution, which allows reversing the role of the null hypothesis to testing a poorly fitting model and then a larger sample provides evidence of good fit. RMSEA is not used to test for perfect fit *ε* = 0 but to test the alternative hypothesis of “close fit” *H*_*a*_ : *ε* ≤ 0.05 or “reasonable fit” *H*_*a*_ : *ε* ≤ 0.08 [26]. Bentler's CFI and the Tucker-Lewis Index (TLI) are relative fit indexes; these measures should be >0.90 [34], and CFI differences of 0.01 between models are considered relevant [35]. The absolute model fit was assessed by calculating the SRMSR between the fatty acids' observed correlations and the correlations predicted by the latent variables; these residuals should be less than 0.10 for a good fitting model [26]. The Schwarz Bayesian Criterion [36], which includes a larger penalty for lack of parsimony than Akaike Information Criteria [37], was also reported. Analyses were performed using SAS software (version 9.2; SAS Institute Inc., Cary, NC) and Mplus (version 6.12; Muthen & Muthen, Los Angeles, CA).

## 3. Results

### 3.1. Exploratory Factor Analysis

Table 1 indicates gender differences in mean levels, in 12 out of 22 RBC fatty acids. The greatest relative differences were higher levels of C16:1 and C18:3n3 in females. The largest absolute differences were that females had about 0.3 percentage point higher and lower levels of C18:2n6 and C22:4n6 than males, respectively. Skewness and kurtosis were calculated for the individual fatty acids, and the following had distributions with an absolute kurtosis index >10 and/or a skew index >3, that is, C20:1, C18:3n3, C20:5n3, and C18:3n6, which became approximately Gaussian using a natural logarithm transformation. However, about 60% of C18:3n6 measurements were <0.1%, which is considered as the reliable detection limit for the GC method, and it appears that the log transformation simply produced normally distributed noise. Therefore, C18:3n6 was excluded from latent variable analysis. Even though univariate normality was reasonable for the individual fatty acids, multivariate normality was rejected (*P* < 0.0001) so robust ML method was implemented in Mplus [28].

Fatty acid concentrations were standardized to produce a correlation matrix (Supplemental Table 2), and the MSA was calculated for each fatty acid and overall (the latter was initially 0.20). The fatty acid with the lowest MSA value was dropped from analysis until all fatty acid MSA values were >0.60. This resulted in the following fatty acids being sequentially excluded from the latent variable analysis: C18:1, C20:1, C18:2n6, C20:2n6, C20:3n6, C24:0, and C24:1. After excluding these fatty acids the overall MSA for the correlation matrix increased to 0.75. Additionally, C22:5n3 needed to be removed from the correlation matrix because it was causing the explained variability in C20:5n3 to be greater than 100%, that is, Heywood condition [38]; hence, 13 fatty acids remained. Afterwards three dimensions were identified for men and women with eigenvalues greater than one.

### 3.2. Confirmatory Factor Analysis

Model M0 allowed intercepts, loadings, and unique variances to be freely estimated for men and women. The absolute fit was good with a SRMSR of 0.035, and the fit was much better than a model with zero correlations since CFI = 0.888 (Table 2). However, the fit measures which adjust for parsimony, that is, RMSEA and TLI, were not near acceptable ranges. In model M1 when the 13 fatty acid means were held constant between gender H_1_ : ***ν***_male_ = ***ν***_female_, the SBC increased by over 400 and the hypothesis was rejected using the chi-squared difference testing between nested models *X*_13_^2^ = 520, *P* < 0.0001. As pointed out earlier since robust ML method was used due to lack of multivariate normality, the chi-squared test statistic was modified [32]. Specifically, to compare model M1 nested in M0, the scaling correction factor was computed as *c*_*d*_ = (DF_M1_∗Scaling_M1_−DF_M0_∗Scaling_M0_)/(DF_M1_ − DF_M0_) = (97∗1.071 − 84∗1.079)/13 = 1.0. Then *X*_13_^2^ = (*X*_M1_^2^∗Scaling_M1_ − *X*_M0_^2^∗Scaling_M0_)/*c*_*d*_ = (2409∗1.071 − 1900∗1.079)/1.019 = 520 (Table 2). All other model fit measures deteriorated as well. These results importantly show that the multivariate fatty acid mean profile was not the same between genders for these fatty acids.

Next the fatty acid correlation structure was tested for gender invariance in multiple steps. When comparing model M2 to M0, H_2_ : Λ_male_ = Λ_female_ we concluded that the factor loadings were different between men and women *X*_30_^2^ = 92, *P* < 0.0001 (Table 2). To test for gender differences in the fatty acids' variances, H_3_ : Θ_male_ = Θ_female_ models M3 and M0 were compared. The fatty acids' variances were* not* different between genders, *X*_13_^2^ = 20.7, respectively, *P* = 0.079. Next the latent variable covariance structure was tested H_4_ : Ψ_male_ = Ψ_female_, by comparing model M2 with M4 and found to be different between men and women (*P* < 0.0001). Likewise the overall covariance structure H_5_ : Σ_male_ = Σ_female  _was different (*P* < 0.0001).

### 3.3. Structural Equation Modeling Constraints

The above results suggested differential fatty acid functioning for men and women, so separate models were developed by gender that allows the standardized latent variables scores to be compared between men and women. Model M3 had a good fit compared to M0 shown by chi-squared difference testing, SRMSR, and CFI; however, the parsimony measures suggest there were still too many parameters. Model M3 is shown for men and women in Supplemental Tables 3 and 4, respectively; the latent variables were named for the fatty acids with the strongest correlations as PUFA, SATURATED, and TRANS FACTORS. When examining the loading estimates between men and women they were quite similar; there were only 3 parameters that differ by >0.10 which included the saturated fatty acids C14:0 and C18:0. These two fatty acids have slightly stronger correlations with the underlying latent variables in women than men. To further reduce the model complexity, constraints were placed on the loading matrix. A threshold of 0.15 was chosen, and parameters were constrained to zero if they had loadings below this threshold.

Correlations among dietary intakes of fatty acids were determined for the subset of 2332 participants with valid food frequency questionnaires [39] (Supplemental Table 5). Dietary intake (g/d) was available for 11 out of 13 RBC fatty acids included in the latent variable model, and C22:4n6 and C22:5n6 were not calculated from the diet. Since RBC fatty acids were correlated with corresponding dietary intakes of fatty acids, covariances were added to the fatty acids residual matrix, Θ, to account for foods being composed of many different fatty acids. Being able to specify which residual covariances to include is a feature unique to structural equation modeling and cannot be accomplished in the context of confirmatory factor analysis. There were 14 strong dietary correlations (*r* > 0.80) that were added to the model. The correlation between C18:1t and C18:2t was extremely high (*r* = 0.98) and caused model convergence issues; therefore, it was subsequently removed.

The biosynthesis process is well known for omega-3 and omega-6 fatty acids [7]. Delta-6 and delta-5 desaturase activity is needed to convert C18:3n3 into C20:5n3 (Figure 1). Then delta-6 desaturase (D6D) is required again to further convert C20:5n3 into C22:6n3. The amount of D6D available for the second conversion to synthesize C22:6n3 may be limited by a function of what is initially consumed to support converting C18:3n3 [40]. Therefore, the amount and variability of both C20:5n3 and C22:6n3 depend (to some extent) on the intake of the parent n3 fatty acid C18:3n3. In the omega-6 fatty acid family, D6D is needed for C22:4n6 to synthesize into C22:5n6. These biochemical steps introduce structural elements into the SEM model, so these three covariances were added to fatty acid residuals matrix Θ. However, omega-3 and omega-6 fatty acids also compete for the desaturase enzymes, and the omega-3 fatty acids are the preferential substrates [6]. So with higher levels of C20:5n3 (whether by biosynthesis or fish oil consumption), D5D activity is inhibited (feedback inhibition, whereby the enzyme senses when enough product has been made and then shuts down). This slows the synthesis of C20:4n6 from C20:3n6. Likewise C22:6n3 and C22:5n6 compete for D6D. These two additional fatty acid covariances were added to the SEM residual matrix as well.

### 3.4. Final Structural Equation Model

After the above constraints were placed on the model, the resulting SEM (Model M6) had a significantly* better* fit than the unrestricted model by gender (M0), *X*_23_^2^ = 506 (*P* < 0.0001). Additionally model M6 was the only model to have a “reasonable” fit with RMSEA <0.08. Also it was the only model to have CFI and TLI > 0.90. Model M6 had a total of 208 − 107 = 101 estimated parameters, including the following gender specific 2 ∗ (23 loadings, 9 means, and 3 latent variable covariances) = 70 and gender invariant (13 residual variances and 13 dietary-related and 5 desaturase-related residual covariances) = 31. The loadings and latent variable correlations (Supplemental Table 6) and fatty acid residual matrix (Supplemental Table 7) are shown for model M6. One loading and three residual covariances were <0.05; these were set to zero for a more parsimonious model (M7). The nested fit between the reduced SEM model M7 was similar to model M6, *X*_4_^2^ = 4.3 (*P* = 0.37) and all the parsimony fit measures (i.e, SBC, RMSEA, and TLI) were improved.

There were four fatty acids with gender mean differences which were not significantly different than zero (i.e, C22:6n3, C20:4n6, C16:0, and C18:1t). The greatest mean differences (all 0.30 to 0.40 SD) between genders were found in two PUFA [ln(C18:3n3) and C22:4n6], one saturated fatty acid [C14:0] and one monounsaturated fatty acid [C16:1]. The loadings, latent variable covariance structure, mean profile, and fatty acid residual matrix for model M7 are shown in Tables 3 and 4. The Mplus code for model M7 is given in Algorithm 1.

## 4. Discussion

Although 22 individual fatty acids were measured during the GC process, 9 were removed from the latent variable analysis because they were not related to the core structure as explained above. However, the individual fatty acids that were removed may still have clinical utility as individual predictor variables. For example, C18:2n6 (Linoleic acid) was fairly independent of the PUFA, SATURATED, and TRANS latent variable scores, and all correlations were around 0.10 (Supplemental Table 8). Since C18:2n6 has been reported inversely related with heart disease [4], it could still have clinical utility as in independent predictor variable in combination with these newly defined latent variables. C22:5n3 (DPA) is intermediate of C20:5n3 (EPA) and C22:6n3 (DHA) in the biosynthesis process (Figure 1) and was the only excluded fatty acid that had a correlation >0.10 with the PUFA FACTOR (*r* = 0.39). However, DPA has less biological activity than the other marine fish oils [41], so it is not anticipated that DPA would be useful as an individual predictor variable of clinical outcomes. Afterwards, the remaining 13 fatty acids were found to be represented by three dimensions, which constituted nearly 70% of the total fatty acid abundance. In the SEM model, the residual variances were equal between genders. Likewise the structural dietary correlations and biosynthesis processes, which were accounted for with residual correlations, did not vary between men and women. The final SEM model fit the data well by all measures.

**Figure 2:**
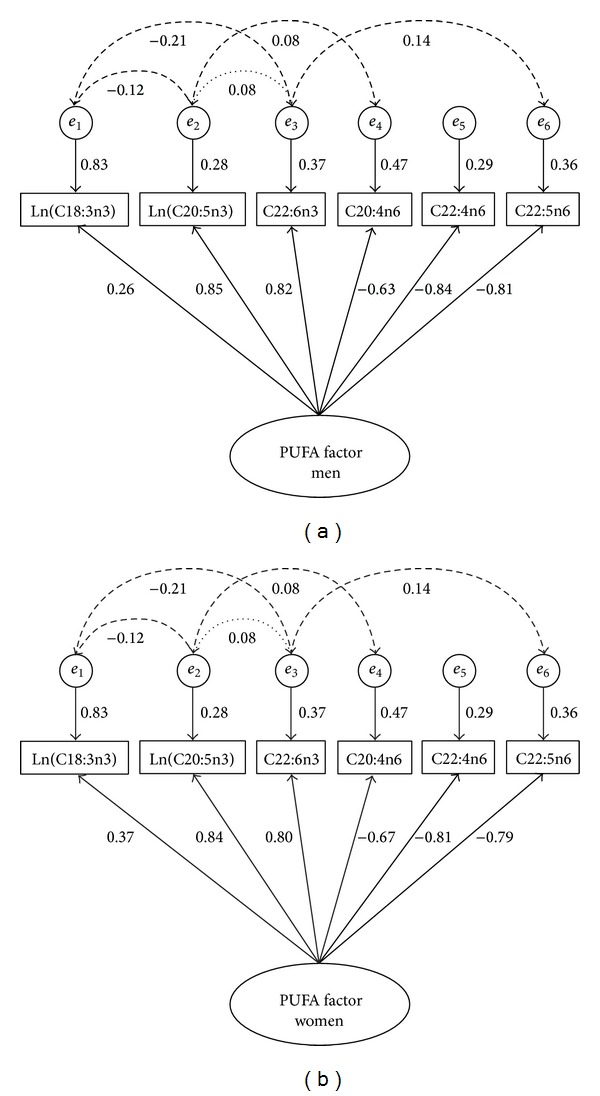
Structural equation model M7 for men (a) and women (b). Solid lines from PUFA FACTOR to fatty acids are gender specific* loadings*, solid lines from circles to fatty acids are residual variances, dotted line indicates structural dietary intake correlation, and dashed lines indicate structural desaturase enzymes required for biosynthesis.

The PUFA FACTOR includes the following fatty acids: ln(C18:3n3), ln(C20:5n3), C22:6n3, C20:4n6, C22:4n6, and C22:5n6 (Figure 2); all of which are found in the PUFA biosynthesis processes shown in Figure 1. The loading directions of the fatty acids included in the PUFA FACTOR are also supported by many of the competing metrics being used in fatty acid research. The omega-3 index is implemented in clinical laboratory testing and is defined as RBC C20:5n3 + C22:6n3 [42]. The omega-3 index was an independent risk factor for all-cause mortality in a study of stable coronary heart disease patients, with higher levels indicating reduced risk [3]. Lower amounts of the omega-3 index were associated with depression in a case-control study of adolescents [43]. In the PUFA FACTOR, for both men and women, these two fatty acids operate in the same direction with similar magnitudes, which supports their summation as a biomarker (although C20:5n3 has been log transformed in the PUFA FACTOR).

The n6/n3 ratio [44], n6 HUFA/total HUFA ratio [45], and C20:4n6/C20:5n3 ratio [46] are all metrics that seek to combine individual fatty acids into more powerful predictors of risk. Although the goal is reasonable, these approaches are criticized as being imprecise and impractical [46]. All of these ratios may be flawed in that the same ratio can be obtained by increasing the numerator or decreasing the denominator, when these fatty acids do not have the same physiological properties. An improvement to these ratios may be the PUFA FACTOR. It is a more nuanced metric since it does not simply add up the masses of different PUFA families and create a ratio; it takes into account the relative strengths of relationship among these linearly “opposing” and interrelated fatty acids and reduces this nexus into a single number. The algorithm for scoring these latent variables from raw fatty acid data is given in the following. 

*Algorithm (algorithm for scoring latent variables)*

*Step 1.* Measure fatty acids as a % of total fatty acids. 

*Step 2.* For C18:3n3 and C20:5n3 transform the values using natural logarithm. 

*Step 3.* Standardize all fatty acids using corresponding overall means and standard deviations from Table 1 into a row vector **Z**_*i*_ (as shown below, the headings indicate the required order). 

*Step 4.* Calculate the latent variable scores **η**_*i*_ for subject *i* using the appropriate male or female matrices as

(1) $$\begin{array}{c} \eta_{i}=\left(Z_{i}-\nu^{T}\right){\left[\Psi \Lambda^{T}{\left(\Lambda \Psi \Lambda^{T}+\Theta \right)}^{-1}\right]}^{T}, \end{array}$$

where ***ν*** is the standardized fatty acid mean column vector given by gender in Table 3. Ψ is the latent variable covariance matrix given by gender in Table 3. Λ is the loading matrix given by gender in Table 3. Θ is the fatty acid residual covariance matrix given in Table 4. *T* means to transpose the matrix. −1 means to take the inverse of the matrix. 

*Example.* Measure RBC fatty acid percent weight composition using gas chromatography as detailed in Harris et al. [41] or similar, then log transform C18:3n3 and C20:5n3, and then standardize all raw data by (value − mean)/SD from Table 1 to produce a row vector:

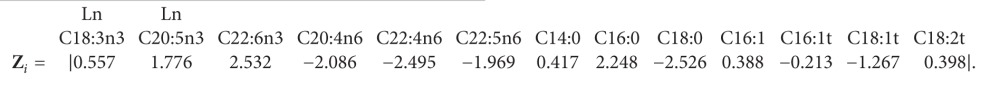
(43)


Lastly the latent variable scores are derived by using  **η**_*i*_ = (**Z**_*i*_ − ***ν*** ^*T*^)[ΨΛ^*T*^(ΛΨΛ^*T*^+Θ)^−1^]^*T*^, with the appropriate vector and matrices used for men or women given in Tables 3 and 4. If the blood sample was from a man or woman, the respective latent variables scores would be

(3) PUFA  SAT  TRANS  FACTORFACTORFACTORηi=|2.771.76−1.56|. or PUFA  SAT  TRANS  FACTORFACTORFACTORηi=|2.561.38−1.52|.

Another approach has been to construct “desaturase ratios” which are based on the known biosynthetic relationships among PUFA [7]. Since it is far too invasive (requiring liver biopsy) to measure the activity of these enzymes directly, they have been estimated empirically by dividing RBC levels of product fatty acids by levels of precursor fatty acids. Thus the delta-6 desaturase (D6D) activity can be estimated by the ratio of 20:3n6/C18:2n6 and the delta-5 desaturase (D5D) activity by the ratio of C20:4n6/C20:3n6 (Figure 1). Interestingly, both of these desaturase ratios have been associated with risk for the development of Type 2 diabetes mellitus in a recent metareview [47]. The ultimate clinical utility of the PUFA FACTOR (versus desaturase or other fatty acid ratios) will be determined in future studies by comparing these metrics as predictors of disease outcomes for mortality, CHD events, development of type 2 diabetes or dementia, and so forth.

## 5. Conclusion

The PUFA FACTOR has much supporting evidence based on fatty acid metabolism and dietary patterns. It was also the first dimension extracted from the data, due to explaining the most variability (about 30% of total in men and women) for these 13 fatty acids. In a previous study these same Framingham subjects were included in a heritability analysis, and it was found that about 25% and 40% of the variance in two of the fatty acids included in the PUFA factor (i.e, EPA and DHA) was due to genetic and environment, respectively [41]. The PUFA factor can also be seen as a unifying theme among the various n3 and n6 metrics typically used in fatty acid research. Since n3 and n6 fatty acids have been implicated in cardiovascular diseases [4, 47], cognitive function [48], brain magnetic resonance imaging [49, 50], depression [43], mortality [1–3], and cellular aging [51] it is reasonable to expect the PUFA FACTOR to have clinical utility for predicting these outcomes. In contrast, the SATURATED and TRANS FACTORS had several cross loadings between them and even include some PUFA. Thus, their interpretations are unclear, which will likely limit their usefulness.

The strengths of this study include a well-characterized structural equation model applied to RBC fatty acid data which incorporates elements of both fatty acid metabolism and dietary intake patterns in defining the model. Additionally the correlation structure of the SEM was decomposed, and the separate components were tested for gender invariance. Another benefit was the use of a large, extensively studied cohort with enrichment for minorities (Framingham). Limitations include that the RBC measurements were from a particular GC method, and since national standards have not been established for measuring fatty acids the sensitivity of these results to other GC methods is unknown. This study measured erythrocytes; other blood fractions or sample types (e.g., whole blood, plasma, and plasma phospholipids) have different rank orders of fatty acid abundances and these may require unique structural equation models. The fit of this SEM needs be tested in independent samples to determine its generalizability beyond the Framingham Study.

## Supplementary Material

The supplementary material includes tables of subject's characteristics, RBC fatty acid and dietary intake correlation matrices, factor correlations with excluded RBC fatty acids, and interim model results.

## Figures and Tables

**Algorithm 1:**
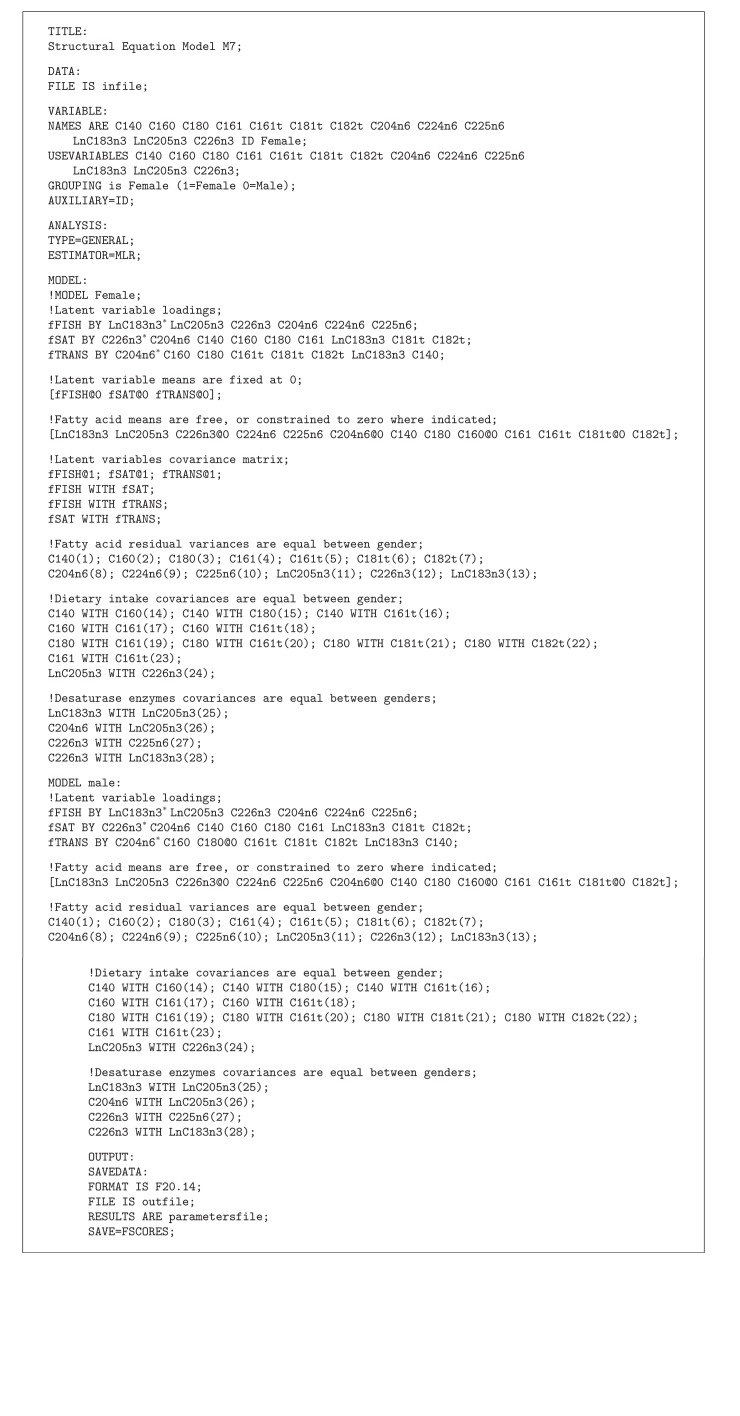
Mplus code for structural equation model M7.

**Table 1:** Framingham subjects' fatty acids; mean (SD).

Fatty acid	Overall *N* = 3196	Male *N* = 1434	Female *N* = 1762	*P* value*
Myristic, C14:0	0.31 (0.08)	0.29 (0.07)	0.32 (0.09)	**<0.0001**
Palmitic, C16:0	21.29 (1.24)	21.24 (1.21)	21.34 (1.26)	0.031
Stearic, C18:0	18.11 (0.95)	18.20 (0.89)	18.04 (1.00)	**<0.0001**
Lignoceric, C24:0	0.43 (0.16)	0.44 (0.16)	0.42 (0.16)	0.012
Palmitoleic, C16:1	0.35 (0.19)	0.31 (0.18)	0.39 (0.19)	**<0.0001**
Oleic, C18:1	13.88 (1.06)	13.90 (1.06)	13.85 (1.06)	0.23
Eicosenoic, C20:1	0.27 (0.11)	0.28 (0.12)	0.27 (0.10)	**<0.0001**
Nervonic, C24:1	0.45 (0.15)	0.46 (0.15)	0.43 (0.15)	**<0.0001**
trans Palmitoleic, C16:1 trans	0.17 (0.05)	0.16 (0.05)	0.17 (0.05)	0.0052
trans Oleic, C18:1 trans	1.62 (0.55)	1.62 (0.57)	1.61 (0.54)	0.56
trans Linoleic, C18:2 trans	0.25 (0.08)	0.24 (0.08)	0.25 (0.08)	**0.0021**
alpha-Linolenic, C18:3n3	0.19 (0.10)^†^	0.17 (0.09)	0.20 (0.11)	**<0.0001**
Eicosapentaenoic (EPA), C20:5n3	0.74 (0.46)^†^	0.71 (0.42)	0.76 (0.49)	**0.0011**
Docosapentaenoic, C22:5n3	2.74 (0.46)	2.80 (0.46)	2.70 (0.45)	**<0.0001**
Docosahexaenoic (DHA), C22:6n3	4.88 (1.38)	4.82 (1.39)	4.92 (1.37)	0.043
Linoleic, C18:2n6	11.19 (1.74)	11.03 (1.63)	11.33 (1.81)	**<0.0001**
gamma-Linolenic, C18:3n6	0.08 (0.09)	0.08 (0.12)	0.09 (0.07)	0.089
Eicosadienoic, C20:2n6	0.28 (0.05)	0.28 (0.05)	0.28 (0.05)	0.23
Eicosatrienoic, C20:3n6	1.59 (0.36)	1.59 (0.36)	1.59 (0.35)	0.66
Arachidonic, C20:4n6	16.78 (1.62)	16.79 (1.57)	16.77 (1.66)	0.66
Docosatetraenoic, C22:4n6	3.76 (0.83)	3.92 (0.83)	3.63 (0.81)	**<0.0001**
Docosapentaenoic, C22:5n6	0.66 (0.19)	0.67 (0.19)	0.64 (0.19)	**<0.0001**

*Two-sample *t*-test, the critical level alpha was set to 0.05/22 = 0.0023 for statistical significance using Bonferroni correction (shown in bold). ^†^Use the following mean (SD) of the log-transformed values when standardizing data as explained in the discussion section for C18:3n3 −6.38 (0.41) and C20:5n3 −5.04 (0.48).

**Table 2:** Goodness-of-fit for testing gender invariance (among 13 fatty acids).

Model constraint (*N* = 3196)	Absolute fit					Relative fit	
	*χ* ^2^/DF^†^	*χ* ^2^ Scaling	SRMSR	SBC	RMSEA upper 90% limit	CFI	TLI
M0: unrestricted by gender	1900/84	1.079	0.035	100055	0.121	0.888	0.791
M1: equal fatty acid means, ***ν***	2409/97	1.071	0.056	100480	0.126	0.857	0.770
M2: equal loadings, Λ	1965/114	1.097	0.040	99919	0.105	0.885	0.843
M3: equal unique Variances, Θ	1673/97	1.255	0.036	99999	0.105	0.902	0.843
M4: equal loadings and latent covariances, Λ,Ψ	1986/120	1.105	0.048	99909	0.102	0.884	0.850
M5: equal fatty acid covariance matrix, Σ	1793/133	1.251	0.050	99853	0.092	0.897	0.879
M6: SEM model	968.8/107	1.217	0.046	98999	0.075	0.947	0.922
M7: reduced SEM M6	967.5/111	1.225	0.046	98972	0.074	0.947	0.925

^†^The total degrees of freedom (DF) = 2 ∗ (91 fatty acid variances/covariances + 13 fatty acid means) = 208 parameters in all models; hence, the number of estimated parameters equals 208 − *X* ^2^ DF; SRMSR = standardized root mean square residual; SBC = Schwarz Bayesian criteria; RMSEA = root mean square error of approximation; CFI = Bentler Comparative Fit Index; TLI = Tucker-Lewis Index.

**Table 3:** Structural equation model M7 factor loadings (Λ) and standardized fatty acid means (***ν***).

Fatty acids	Men				Women			
	PUFA*	SAT	TRANS	Mean	PUFA*	SAT	TRANS	Mean
Ln(C18:3n3)	0.257	0.138	0.070	−0.186	0.370	0.149	0.114	0.149
Ln(C20:5n3)	0.847	0	0	−0.043	0.844	0	0	0.036
C22:6n3	0.815	−0.373	0	0	0.801	−0.335	0	0
C20:4n6	−0.634	−0.289	−0.215	0	−0.667	−0.285	−0.204	0
C22:4n6	−0.837	0	0	0.168	−0.814	0	0	−0.138
C22:5n6	−0.806	0	0	0.083	−0.788	0	0	−0.069
C14:0	0	0.633	0.056	−0.221	0	0.781	0.105	0.178
C16:0	0	0.754	−0.227	0	0	0.808	−0.290	0
C18:0	0	−0.524	0	0.072	0	−0.681	0.084	−0.054
C16:1	0	0.723	0	−0.209	0	0.812	0	0.169
C16:1t	0	0	0.499	−0.070	0	0	0.540	0.057
C18:1t	0	−0.114	0.888	0	0	−0.168	0.843	0
C18:2t	0	0.248	0.728	−0.065	0	0.239	0.738	0.053

	Factor correlations (Ψ)							
Factor								
PUFA	1		0.190	−0.323	1		0.249	−0.385
SAT	0.190		1	−0.160	0.249		1	0.003
TRANS	−0.323		−0.160	1	−0.385		0.003	1

*Direction of signs is arbitrary; the signs for the PUFA loadings and factor correlations have been reversed in this table to make more n3 fatty acid positively associated with the PUFA FACTOR.

**Table 4:** Structural equation model M7 fatty acid residuals matrix (Θ).

	Ln C18:3n3	Ln C20:5n3	C22:6n3	C20:4n6	C22:4n6	C22:5n6	C14:0	C16:0	C18:0	C16:1	C16:1t	C18:1t	C18:2t
Ln(C18:3n3)	0.832	−0.120*	−0.214*	0	0	0	0	0	0	0	0	0	0
Ln(C20:5n3)	−0.120*	0.278	0.077**	0.081*	0	0	0	0	0	0	0	0	0
C22:6n3	−0.214*	0.077**	0.372	0	0	0.137*	0	0	0	0	0	0	0
C20:4n6	0	0.081*	0	0.466	0	0	0	0	0	0	0	0	0
C22:4n6	0	0	0	0	0.290	0	0	0	0	0	0	0	0
C22:5n6	0	0	0.137*	0	0	0.358	0	0	0	0	0	0	0
C14:0	0	0	0	0	0	0	0.437	−0.066**	0.096**	0	0.090**	0	0
C16:0	0	0	0	0	0	0	−0.066**	0.290	0	0.034**	−0.115**	0	0
C18:0	0	0	0	0	0	0	0.096**	0	0.617	−0.080**	−0.081**	−0.219**	−0.082**
C16:1	0	0	0	0	0	0	0	0.034**	−0.080**	0.360	−0.099**	0	0
C16:1t	0	0	0	0	0	0	0.090**	−0.115**	−0.081**	−0.099**	0.720	0	0
C18:1t	0	0	0	0	0	0	0	0	−0.219**	0	0	0.220	0
C18:2t	0	0	0	0	0	0	0	0	−0.082**	0	0	0	0.426

**indicates dietary intake-related covariances; *indicates biosynthesis-related covariances; fatty acid residual variances are shown on the main diagonal.
