# Supplementary material for: Structural Equation Modeling for Analyzing Erythrocyte Fatty Acids in Framingham
Source: Comput Math Methods Med. 2014 Apr 15;2014:160520. doi: 10.1155/2014/160520 (PMC4052884; doi:10.1155/2014/160520)
Supplement: Supplementary file 1 — The supplementary material includes tables of subject's characteristics, RBC fatty acid and dietary intake correlation matrices, factor correlations with excluded RBC fatty acids, and interim model results. [file 160520.f1.pdf]

Table 1: Framingham Subjects' Characteristics ($N = 3196$).

Variable [% missing][a]	Mean (SD) or n (%)
Age (years)	66 (9)
Female	1762 (55%)
White	2899 (91%)
Highest Education:	
Grades < 11	136 (4%)
Grade 12+	1485 (47%)
Associate/Bachelor	927 (29%)
Graduate or Professional	617 (19%)
Systolic (mm Hg)	128 (17)
Diastolic (mm Hg)	74 (10)
Heart Rate (bpm)	62 (10)
Hypertension Treatment	1566 (49%)
Waist Girth at Umbilicus (inches)	40.0 (5.8)
BMI (kg/m^2)	28.4 (5.5)
1-2 Drinks/Day[b] [1.3%]	414 (13%)
Physical Activity Index [1.1%]	35.2 (5.3)
Current Smoker	270 (8%)
LDL Cholesterol (mg/dL)	105 (32)
HDL Cholesterol (mg/dL)	57 (18)
Triglycerides (mg/dL)	118 (71)
Lipid Pharmacotherapy	1373 (43%)
Fish Oil Supplementation	322 (10%)
Aspirin (3+/week)	1383 (43%)
Glucose (mg/dL)	107 (24)
Hemoglobin A1c %	5.8 (0.7)
Diabetes	462 (14%)
Urine Albumin/Creatinine (mg/g) [1.7%]	0.26 (1.29)
Prevalent Cardiovascular Disease	498 (16%)
Prevalent Congestive Heart Failure	77 (2%)
Prevalent Coronary Heart Disease	326 (10%)

[a]All variables had <1% missing data unless noted otherwise; [b]Indicator variable for females >7 drinks/week, males >14 drinks/week.
