# Supplementary material for: Structural Equation Modeling for Analyzing Erythrocyte Fatty Acids in Framingham
Source: Comput Math Methods Med. 2014 Apr 15;2014:160520. doi: 10.1155/2014/160520 (PMC4052884; doi:10.1155/2014/160520)
Supplement: Supplementary file 2 [file 160520.f2.pdf]

Table 2: RBC Fatty Acid Correlations (N = 3196).

	Saturated				Monounsaturated				Trans			Omega-3 Polyunsaturated				Omega-6 Polyunsaturated						
	C14:0	C16:0	C18:0	C24:0	C16:1	C18:1	C20:1	C24:1	C16:1t	C18:1t	C18:2t	C18:3	C20:5	C22:5	C22:6	C18:2	C18:3	C20:2	C20:3	C20:4	C22:4	C22:5
C14:0	1.0	—	—	—	—	—	—	—	—	—	—	—	—	—	—	—	—	—	—	—	—	—
C16:0	0.49**	1.0	—	—	—	—	—	—	—	—	—	—	—	—	—	—	—	—	—	—	—	—
C18:0	−0.36**	−0.51**	1.0	—	—	—	—	—	—	—	—	—	—	—	—	—	—	—	—	—	—	—
C24:0	−0.16	−0.28	−0.16	1.0	—	—	—	—	—	—	—	—	—	—	—	—	—	—	—	—	—	—
C16:1	0.59**	0.66***	−0.56**	−0.10	1.0	—	—	—	—	—	—	—	—	—	—	—	—	—	—	—	—	—
C18:1	0.11	0.20	−0.38**	0.08	0.32**	1.0	—	—	—	—	—	—	—	—	—	—	—	—	—	—	—	—
C20:1†	−0.17	−0.18	−0.07	0.13	−0.05	0.15	1.0	—	—	—	—	—	—	—	—	—	—	—	—	—	—	—
C24:1	−0.16	−0.36**	−0.05	0.84*	−0.18	−0.01	0.04	1.0	—	—	—	—	—	—	—	—	—	—	—	—	—	—
C16:1t	0.12	−0.28	−0.04	−0.06	−0.12	−0.03	0.03	−0.05	1.0	—	—	—	—	—	—	—	—	—	—	—	—	—
C18:1t	−0.07	−0.38**	−0.05	−0.18	−0.19	−0.06	0.00	−0.10	0.45**	1.0	—	—	—	—	—	—	—	—	—	—	—	—
C18:2t	0.21	−0.04	−0.17	−0.15	0.14	0.06	−0.11	−0.12	0.42**	0.58**	1.0	—	—	—	—	—	—	—	—	—	—	—
C18:3n3†	0.26	0.14	−0.20	0.03	0.13	0.00	−0.20	0.03	−0.01	−0.01	0.12	1.0	—	—	—	—	—	—	—	—	—	—
C20:5n3†	0.12	0.24	−0.08	0.05	0.09	0.02	−0.06	0.00	−0.08	−0.34**	−0.19	0.15	1.0	—	—	—	—	—	—	—	—	—
C22:5n3	−0.08	0.00	0.17	−0.03	−0.13	−0.09	−0.07	−0.03	−0.02	−0.13	−0.16	0.01	0.64***	1.0	—	—	—	—	—	—	—	—
C22:6n3	−0.15	0.00	0.03	0.15	−0.18	−0.08	−0.02	0.08	−0.07	−0.26	−0.25	−0.02	0.70***	0.41**	1.0	—	—	—	—	—	—	—
C18:2n6	0.13	−0.02	−0.26	−0.06	0.00	−0.16	0.06	−0.03	0.13	0.12	0.14	0.35**	−0.14	−0.32**	−0.25	1.0	—	—	—	—	—	—
C18:3n6†	0.30**	0.25	−0.29	−0.04	0.38**	0.12	−0.10	−0.02	−0.04	−0.03	0.12	0.08	−0.01	−0.14	−0.19	0.06	1.0	—	—	—	—	—
C20:2n6	−0.13	−0.09	−0.05	0.08	−0.12	−0.16	0.17	−0.03	−0.11	0.05	0.01	0.21	−0.20	−0.17	−0.07	0.45**	−0.03	1.0	—	—	—	—
C20:3n6	0.19	0.12	−0.11	−0.02	0.23	0.03	0.02	0.00	−0.01	0.03	0.16	0.10	−0.20	−0.16	−0.22	0.25	0.10	0.24	1.0	—	—	—
C20:4n6	−0.26	−0.39**	0.31**	0.12	−0.24	−0.27	−0.04	0.18	0.00	0.07	−0.10	−0.32**	−0.44**	−0.21	−0.33**	−0.47**	−0.04	−0.22	−0.31**	1.0	—	—
C22:4n6	−0.21	−0.29	0.25	−0.07	−0.18	−0.19	0.09	−0.03	0.03	0.26	0.07	−0.30**	−0.69***	−0.25	−0.59**	−0.24	−0.02	0.01	−0.03	0.58**	1.0	—
C22:5n6	−0.05	−0.20	0.05	0.05	0.01	−0.09	0.06	0.08	0.06	0.24	0.11	−0.24	−0.70***	−0.40**	−0.48**	−0.19	−0.02	−0.04	0.15	0.50**	0.67***	1.0

†Natural logarithm transformation; Cells are shaded by strength of simple correlation, $^{**}0.30 \le |r| < 0.60$. $^{***}0.60 \le |r| < 0.80$. $^{*}0.80 \le |r|$.
