# Supplementary material for: Structural Equation Modeling for Analyzing Erythrocyte Fatty Acids in Framingham
Source: Comput Math Methods Med. 2014 Apr 15;2014:160520. doi: 10.1155/2014/160520 (PMC4052884; doi:10.1155/2014/160520)
Supplement: Supplementary file 3 [file 160520.f3.pdf]

Table 3: Model M3 for Men.

Fatty Acids	Quartimin Rotated Factor Loadings			Residual Variances
	PUFA	SAT	TRANS	
Ln(C18:3n3)	−0.187**	0.167**	0.170**	0.855
Ln(C20:5n3)	−0.824**	0.023	−0.119	0.254
C22:6n3	−0.751**	−0.305**	−0.147	0.373
C20:4n6	0.601**	−0.290**	−0.218**	0.495
C22:4n6	0.854**	−0.102	−0.062	0.253
C22:5n6	0.789**	0.024	−0.022	0.392
C14:0	−0.014	0.557**	0.075	0.532
C16:0	−0.104	0.775**	−0.186**	0.267
C18:0	0.088	−0.545**	−0.216**	0.551
C16:1	0.116	0.787**	−0.064	0.261
C16:1t	−0.067	−0.148	0.534**	0.653
C18:1t	0.078	−0.186**	0.824**	0.282
C18:2t	−0.006	0.158**	0.734**	0.414
Factor	Factor Correlations			
SAT	−0.106	1	. . .	
TRANS	0.198	−0.075	1	
Extracted Cumulative Proportion of Variances				
Common	0.26	0.43	0.55	
Total	0.29	0.49	0.64	

** loadings >0.15.
