# Supplementary material for: Structural Equation Modeling for Analyzing Erythrocyte Fatty Acids in Framingham
Source: Comput Math Methods Med. 2014 Apr 15;2014:160520. doi: 10.1155/2014/160520 (PMC4052884; doi:10.1155/2014/160520)
Supplement: Supplementary file 4 [file 160520.f4.pdf]

Table 4: Model M3 for Women.

Fatty Acids	Quartimin Rotated Factor Loadings PUFA	SAT	TRANS	Residual Variances
Ln(C18:3n3)	−0.281**	0.190**	0.175**	0.855
Ln(C20:5n3)	−0.839**	−0.024	−0.096	0.254
C22:6n3	−0.740**	−0.258**	−0.114	0.373
C20:4n6	0.605**	−0.322**	−0.194**	0.495
C22:4n6	0.801**	−0.145	−0.042	0.253
C22:5n6	0.772**	0.040	0.012	0.392
C14:0	−0.040	0.684**	0.178**	0.532
C16:0	−0.060	0.822**	−0.262**	0.267
C18:0	0.080	−0.686**	−0.168**	0.551
C16:1	0.081	0.882**	−0.016	0.261
C16:1t	−0.107	−0.137	0.621**	0.653
C18:1t	0.136	−0.182**	0.757**	0.282
C18:2t	0.039	0.182**	0.749**	0.414
Factor	Factor Correlations			
SAT	−0.198	1	⋯	
TRANS	0.232	0.030	1	
Extracted Cumulative Proportion of Variances				
Common	0.28	0.46	0.57	
Total	0.31	0.52	0.66	

**loadings >0.15.
