# Supplementary material for: Structural Equation Modeling for Analyzing Erythrocyte Fatty Acids in Framingham
Source: Comput Math Methods Med. 2014 Apr 15;2014:160520. doi: 10.1155/2014/160520 (PMC4052884; doi:10.1155/2014/160520)
Supplement: Supplementary file 5 [file 160520.f5.pdf]

TABLE 5: Fatty Acid Dietary Intake Descriptive Statistics and Correlations ($N = 2332$).

| Fatty Acid | C14:0 | C16:0 | C18:0 | C16:1 | C16:1t | C18:1t | C18:2t | C18:3 | C20:5 | C22:6 | C20:4 |
|---|---|---|---|---|---|---|---|---|---|---|---|
| Mean [g/d] | 1.95 | 12.89 | 5.96 | 1.28 | 0.09 | 2.00 | 0.49 | 1.28 | 0.13 | 0.21 | 0.14 |
| Std. Dev. | 1.05 | 5.51 | 2.74 | 0.60 | 0.05 | 1.14 | 0.27 | 0.67 | 0.14 | 0.18 | 0.07 |
| Skewness | 1.9 | 1.2 | 1.3 | 1.4 | 1.6 | 1.7 | 1.6 | 4.4 | 2.8 | 2.8 | 1.6 |
| Kurtosis | 6.7 | 2.6 | 3.0 | 4.1 | 4.8 | 4.2 | 3.9 | 45.7 | 20.2 | 16.0 | 7.6 |
| Mean† | — | — | — | — | — | — | — | 0.15 | −2.54 | −1.87 | — |
| Std. Dev. | — | — | — | — | — | — | — | 0.44 | 1.07 | 0.83 | — |
| Skewness | — | — | — | — | — | — | — | 0.2 | −0.2 | −0.5 | — |
| Kurtosis | — | — | — | — | — | — | — | 0.8 | −0.7 | 0.7 | — |
| C14:0 | 1.00 | — | — | — | — | — | — | — | — | — | — |
| C16:0 | 0.88** | 1.00 | — | — | — | — | — | — | — | — | — |
| C18:0 | 0.84** | 0.97** | 1.00 | — | — | — | — | — | — | — | — |
| C16:1 | 0.77 | 0.90** | 0.83** | 1.00 | — | — | — | — | — | — | — |
| C16:1t | 0.94** | 0.90** | 0.88** | 0.84** | 1.00 | — | — | — | — | — | — |
| C18:1t | 0.61 | 0.78 | 0.83** | 0.62 | 0.66 | 1.00 | — | — | — | — | — |
| C18:2t | 0.68 | 0.81** | 0.85** | 0.61 | 0.68 | 0.98** | 1.00 | — | — | — | — |
| C18:3n3† | 0.52 | 0.70 | 0.64 | 0.58 | 0.51 | 0.53 | 0.57 | 1.00 | — | — | — |
| C20:5n3† | 0.03 | 0.04 | −0.02 | 0.14 | −0.02 | −0.07 | −0.06 | 0.17 | 1.00 | — | — |
| C22:6n3† | 0.07 | 0.09 | 0.03 | 0.22 | 0.01 | −0.05 | −0.03 | 0.23 | 0.94** | 1.00 | — |
| C20:4n6 | 0.34 | 0.53 | 0.45 | 0.69 | 0.35 | 0.29 | 0.28 | 0.44 | 0.33 | 0.46 | 1.00 |

†Natural logarithm transformation; **Strong correlations ($r > 0.80$).
