# Supplementary material for: Structural Equation Modeling for Analyzing Erythrocyte Fatty Acids in Framingham
Source: Comput Math Methods Med. 2014 Apr 15;2014:160520. doi: 10.1155/2014/160520 (PMC4052884; doi:10.1155/2014/160520)
Supplement: Supplementary file 6 [file 160520.f6.pdf]

Table 6: Structural Equation Model M6 Factor Loadings, $\mathbf{\Lambda}$.

Fatty Acids	Men			Women		
	PUFA	SAT	TRANS	PUFA	SAT	TRANS
Ln(C18:3n3)	−0.251	0.142	0.069	−0.364	0.154	0.113
Ln(C20:5n3)	−0.838	0	0	−0.834	0	0
C22:6n3	−0.812	−0.382	0	−0.803	−0.347	0
C20:4n6	0.631	−0.286	−0.209	0.661	−0.284	−0.197
C22:4n6	0.846	0	0	0.822	0	0
C22:5n6	0.815	0	0	0.797	0	0
C14:0	0	0.635	0.055	0	0.782	0.106
C16:0	0	0.736	−0.227	0	0.792	−0.288
C18:0	0	−0.495	−0.016	0	−0.654	0.063
C16:1	0	0.718	0	0	0.805	0
C16:1t	0	0	0.496	0	0	0.538
C18:1t	0	−0.121	0.890	0	−0.178	0.846
C18:2t	0	0.243	0.723	0	0.232	0.736
Factor	Factor Correlations, $\mathbf{\Psi}$					
SAT	−0.186	1	⋯	−0.255	1	⋯
TRANS	0.317	−0.151	1	0.380	0.013	1
