# Supplementary material for: Structural Equation Modeling for Analyzing Erythrocyte Fatty Acids in Framingham
Source: Comput Math Methods Med. 2014 Apr 15;2014:160520. doi: 10.1155/2014/160520 (PMC4052884; doi:10.1155/2014/160520)
Supplement: Supplementary file 7 [file 160520.f7.pdf]

Table 7: Structural Equation Model M6 Fatty Acid Residuals Matrix, $\mathbf{\Theta}$.

	Ln C18:3n3	Ln C20:5n3	C22:6n3	C20:4n6	C22:4n6	C22:5n6	C14:0	C16:0	C18:0	C16:1	C16:1t	C18:1t	C18:2t
Ln(C18:3n3)	0.834	-0.114^{*}	-0.209^{*}	0	0	0	0	0	0	0	0	0	0
Ln(C20:5n3)	-0.114^{*}	0.294	0.089^{**}	0.073^{*}	0	0	0	0	0	0	0	0	0
C22:6n3	-0.209^{*}	0.089^{**}	0.372	0	0	0.142^{*}	0	0	0	0	0	0	0
C20:4n6	0	0.073^{*}	0	0.469	0	0	0	0	0	0	0	0	0
C22:4n6	0	0	0	0	0.275	-0.020^{*}	0	0	0	0	0	0	0
C22:5n6	0	0	0.142^{*}	0	-0.020^{*}	0.343	0	0	0	0	0	0	0
C14:0	0	0	0	0	0	0	0.434	-0.054^{**}	0.080^{**}	0	0.087^{**}	0	0
C16:0	0	0	0	0	0	0	-0.054^{**}	0.321	-0.045^{**}	0.054^{**}	-0.120^{**}	0	0.003^{**}
C18:0	0	0	0	0	0	0	0.080^{**}	-0.045^{**}	0.654	-0.107^{**}	-0.065^{**}	-0.198^{**}	-0.074^{**}
C16:1	0	0	0	0	0	0	0	0.054^{**}	-0.107^{**}	0.369	-0.104^{**}	0	0
C16:1t	0	0	0	0	0	0	0.087^{**}	-0.120^{**}	-0.065^{**}	-0.104^{**}	0.722	0	0
C18:1t	0	0	0	0	0	0	0	0	-0.198^{**}	0	0	0.213	0
C18:2t	0	0	0	0	0	0	0	0.003^{**}	-0.074^{**}	0	0	0	0.429

**indicates dietary intake-related covariances; *indicates biosynthesis-related covariances; Fatty acid residual variances are shown on the main diagonal.
