# Supplementary material for: Structural Equation Modeling for Analyzing Erythrocyte Fatty Acids in Framingham
Source: Comput Math Methods Med. 2014 Apr 15;2014:160520. doi: 10.1155/2014/160520 (PMC4052884; doi:10.1155/2014/160520)
Supplement: Supplementary file 8 [file 160520.f8.pdf]

TABLE 8: Spearman Correlations Between Latent Variable Scores and Excluded Fatty Acids.

	PUFA FACTOR	SATURATED FACTOR	TRANS FACTOR
Lignoceric, C24:0	0.045	−0.169	−0.269
Oleic, C18:1	0.097	0.275	−0.084
Eicosenoic, C20:1	−0.088	−0.139	−0.071
Nervonic, C24:1	−0.004	−0.232	−0.185
Docosapentaenoic, C22:5n3	0.393	−0.026	−0.130
Linoleic, C18:2n6	0.100	0.095	0.110
gamma-Linolenic, C18:3n6	0.008	0.418	−0.015
Eicosadienoic, C20:2n6	0.006	−0.104	0.018
Eicosatrienoic, C20:3n6	−0.067	0.249	0.117
